# ﻿Review of the genus *Isca* (Ephemeroptera, Leptophlebiidae) from China with a new species and a new species record

**DOI:** 10.3897/zookeys.1234.140905

**Published:** 2025-04-11

**Authors:** De-Wen Gong, Chang-Fa Zhou

**Affiliations:** 1 College of Life Sciences, Nanjing Normal University, Nanjing 210023, China Nanjing Normal University Nanjing China

**Keywords:** Barcode gene, China, COI, mayfly, morphology, new species, subgenus, taxonomy

## Abstract

Previously, only one *Isca* species (*Iscapurpurea* Gillies, 1951) was recorded in China. In this review, three *Isca* species are presented and photographed. Among them, *I.acutata***sp. nov.** is a new species with an acute male penal apex, a uniform brown abdomen of the imago, and a bilamellate gill VII of the nymph. Another species, *I.fascia*, previously reported from Vietnam, is found in China for the first time. The nymphs of *I.fascia* exhibit a banded body and a convex apex of the penes. The structure of the third species *I.purpurea*, shown graphically, has separated penes and a paler nymphal body than the other two species. The diverse morphology of the penes, wings, venation, and gills show that *Isca* has diverse species and evolutionary directions, suggesting that the previous subgeneric classification may not adequately represent some species. Biologically, their nymphs were observed living in tiny pits on substrate surfaces, where their ventral gills may facilitate respiration while they hide.

## ﻿Introduction

The genus *Isca* was first established by Gillies in 1951, based on imaginal specimens of *Iscapurpurea* Gillies, 1951, collected from southern China (Hong Kong) and India. Later, Peters and Edmunds (1970) associated the nymphs with the imago of this genus. In the same paper, they also established two subgenera for two additional species: I. (Minyphlebia) janiceae Peters & Edmunds, 1970 from Thailand and I. (Tanycola) serendiba Peters & Edmunds, 1970 from Ceylon, based on wing characteristics (such as the presence or absence of wing cilia, the fusion or unfusion of MP2 with MP1, and whether MA forks were more or less than halfway from the base to the margin). Nguyen and Bae (2003) named the fourth species *I.fascia* from Vietnam with both nymphal and imaginal stages. The fifth species *I.lea*, described by Sartori and Derleth (2010) from Indonesia (East Kalimantan) based on the nymphal stage, was the only one with a bilamellate gill VII. Despite being one of the first countries noted for the presence of *Isca*, further research on the Chinese species has not been conducted.

Both adults and nymphs of the known *Isca* species show high morphological diversity. Their wings can be transparent (*I.janiceae*) or translucent (*I.purpurea*, *I.serendiba*, *I.fascia*), with marginal cilia (*I.purpurea*, *I.serendiba*, *I.fascia*) or without (*I.janiceae*), nymphs with bilamellate gills VII (*I.lea*) or unilamellate gills VII (*I.purpurea*, *I.serendiba*, *I.fascia*, *I.janiceae*). The increasing number of reported species suggests a broader variety of potential evolutionary trends within the genus.

Both nymphs and imagoes of *Isca* have remarkable characters. Besides their tiny bodies, the imagoes have forewings only, and the nymphs have ventral gills. To our knowledge, only psammophilous nymphs in the genera *Paradolania* Zhou, 2024, *Dolania* Edmunds & Traver, 1959, and *Behningia* Lestage, 1930 exhibit similar ventral gills (Zheng et al. 2024), while some species in the family Oligoneuriidae possess either ventral gill I or gills I–II (Bauernfeind and Soldán 2012). However, *Isca* nymphs have been reported living on stony substrates (Peters and Edmunds 1970). Further biological observations of these species will unveil the function of their morphological characters.

In recent years, we conducted extensive collection efforts across South China to uncover more leptophlebiids in this region. As a result, three *Isca* species were found, including the known one *I.purpurea*, the newly recorded *I.fascia* and a new species *I.acutata* sp. nov. These findings highlight the diversity of the *Isca* genus in China.

## ﻿Material and methods

### ﻿Collecting

The nymphs were collected by hand net in a little stream near the road, some of the adults were caught under the leaves or on the spider webs nearby, and the rest of them were reared indoors from mature nymphs. All materials were stored in ethanol (more than 80%).

### ﻿Observing

Digital photos were taken using a Sony A7RIV Interchangeable Lens Digital Camera with a Laowa FF 25 mm F2.8 Ultra Macro (macro lens), and a Nikon Eclipse 50i microscope with an Mshot MDX10 Image System. Final plates were prepared and polished using Adobe Photoshop 2022.

### ﻿SEM

Eggs were dissected from the female adult (*I.acutata* sp. nov.) and mature female nymphs (*I.purpurea*). All SEM (scanning electronic microscope) samples were prepared with a standard protocol: fixed in 4% glutaraldehyde for 5–8 h, rinsed with PBS (physiological saline) 2 or 3 times (10–15 min each), dehydrated in concentration gradient acetone (30%, 50%, 70%, 80%, 90%, 100%, 10 to 15 min each), and coated with gold film in a vacuum.

### ﻿COI sequencing

In order to associate our nymphs and adults of selected species and differentiate species of *Isca*, we sequenced five Chinese individuals. Morphologically, three of them were *Iscapurpurea*, and two were *I.acutata* sp. nov. Additionally, we downloaded three Indian *I.purpurea* sequences from GenBank and used the species *Habrophlebiodeszijinensis* as the outgroup (Table 1).

**Table 1.** The species and their COI sequences used in this research.

Species	GenBank accession number	Remarks
*I.purpurea* (1)	PQ558934	China (Fujian), Male
*I.purpurea* (2)	PQ558935	China (Fujian), Female
*I.purpurea* (3)	PQ558936	China (Guangdong)
*I.acutata* sp. nov. (1)	PQ558937	China (Hainan) Nymph
*I.acutata* sp. nov. (2)	PQ558938	China (Hainan) Male
*I.purpurea* (4)	ON557588	India
*I.purpurea* (5)	MW160191	India
*I.purpurea* (6)	LC061468	India (Selvakumar et al. 2016)
*Habrophlebiodeszijinensis*	OP908297	–

The DNA extraction and ampliﬁcation followed the process by Zheng and Zhou (2021). The COI sequence data of these species was uploaded to GenBank, and its GenBank accession number is listed in Table 1. The sequences were aligned with the software ClustalW.

All specimens used in this study are deposited in the Mayfly Collection, College of Life Sciences, Nanjing Normal University (NNU).

### ﻿Nymph-adult associating

The association between nymph and imago of *I.acutata* sp. nov. and *I.purpurea* was established using CO1 sequences (see Tables 1, 2). At the same time, one male of *I.acutata* sp. nov. was reared from nymphs, and both imaginal and mature nymphal genitalia were compared and associated with three species in this study. Furthermore, the color pattern of their nymphs and adults, especially those of *I.fascia*, were good characters in our association.

**Table 2.** K2P genetic distance among the COI sequences.

	*I.purpurea* (1)	*I.purpurea* (2)	*I.purpurea* (3)	*I.acutata* sp. nov. (1)	*I.acutata* sp. nov. (2)	*I.purpurea* (4)	*I.purpurea* (5)
*I.purpurea* (2)	0.29%						
*I.purpurea* (3)	1.63%	1.94%					
*I.acutata* sp. nov. (1)	20.01%	20.11%	20.39%				
*I.acutata* sp. nov. (2)	20.79%	20.57%	20.79%	0.48%			
*I.purpurea* (4)	19.61%	20.12%	19.42%	17.61%	18.13%		
*I.purpurea* (5)	19.07%	19.57%	18.88%	17.07%	17.56%	1.05%	
*I.purpurea* (6)	16.68%	16.78%	16.47%	18.71%	18.44%	12.63%	11.81%

To reconstruct the molecular tree, Bayesian inference (BI) and maximum likelihood (ML) analyses were performed. The partition model was selected by ModelFinder based on BIC (Bayesian information criterion) and AICc (standard correction to Akaike information criterion) (Kalyaanamoorthy et al. 2017). BI phylogenies were reconstructed using MrBayes v. 3.2.6 through the online CIPRES Science Gateway (Miller et al. 2011; Ronquist et al. 2012), with the following settings: two parallel runs with four Markov chains were run for 10 million generations (with a sampling frequency of 1000), with a burn-in of 25% trees. RAxML v. 8.2.0 was used for ML analyses, with the model GTRGAMMAI and 1000 bootstrap replicates (Stamatakis 2014). FigTree v. 1.4.2 (http://tree.bio.ed.ac.uk/software/figtree/; accessed on 5 July 2021) was used for the editing of phylogenetic trees.

## ﻿Results

### ﻿Taxonomy

**Genus *Isca* Gillies, 1951**

Figs 1–6, 8–15

#### ﻿ Isca acutata sp. nov.

Taxon classificationAnimaliaEphemeropteraLeptophlebiidae

BDFDA142-B505-5672-B164-DBF20FF90CCF

https://zoobank.org/13349861-975E-460C-917E-CEC50D0F32F3

**Figure 1.**
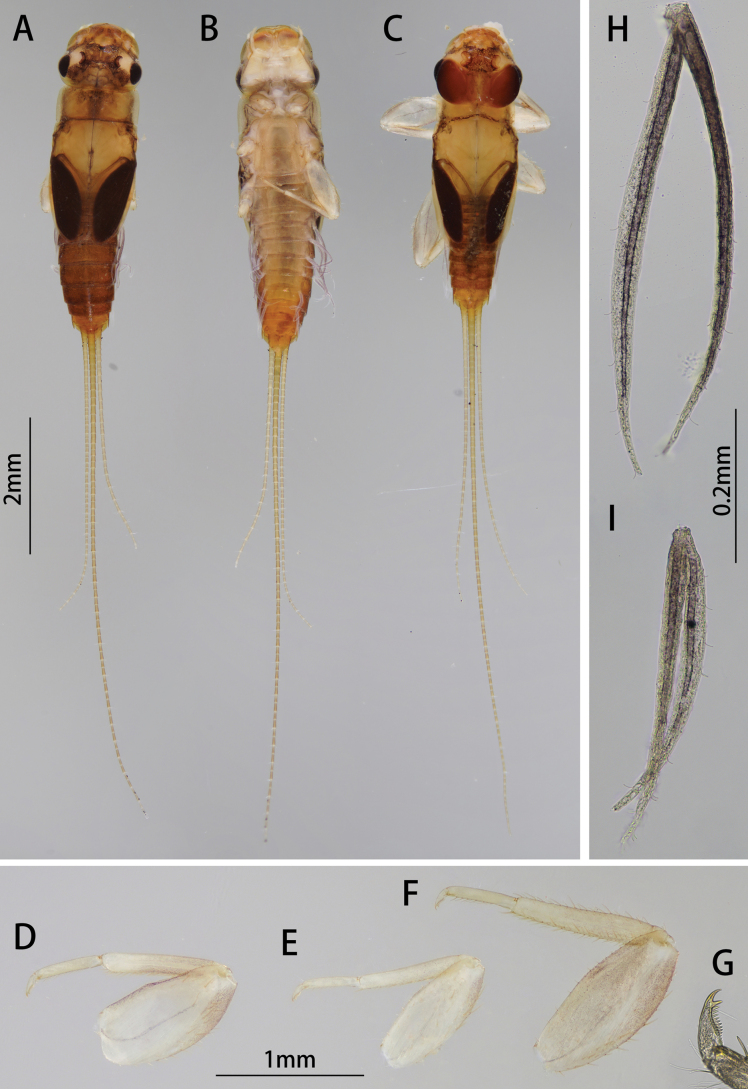
Nymphal structures of *I.acutata* sp. nov.: **A** female nymph (dorsal view) **B** female nymph (ventral view) **C** male nymph (dorsal view) **D** foreleg **E** midleg **F** hindleg **G** claw **H** gill IV **I** gill VII.

**Figure 2.**
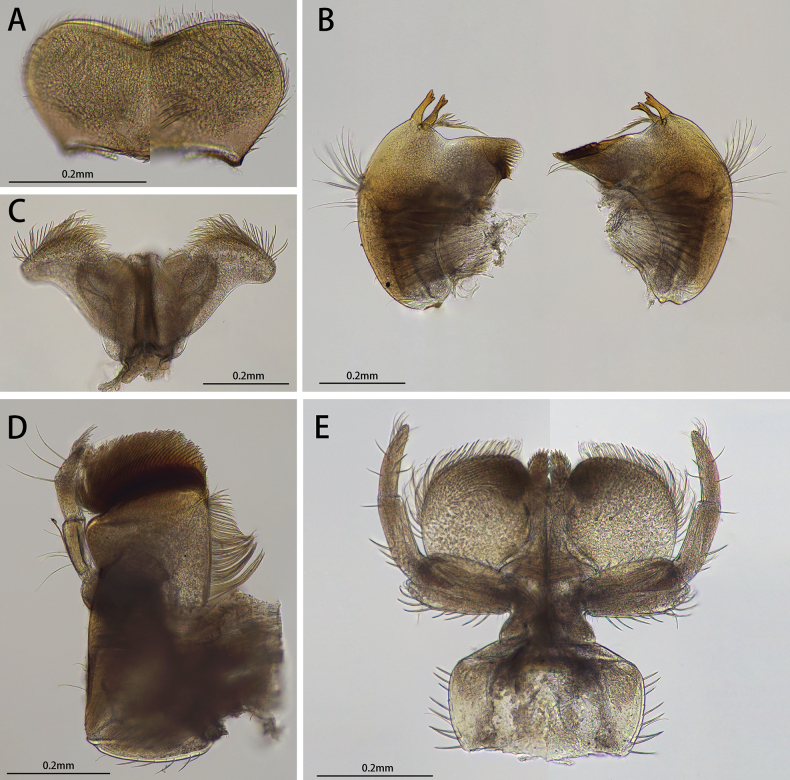
Mouth part of *I.acutata* sp. nov.: **A** labrum (left: dorsal view, right: ventral view) **B** mandible (left: left mandible / right: right mandible) **C** hypopharynx **D** maxilla (ventral view) **E** labium (left: dorsal view / right: ventral view).

**Figure 3.**
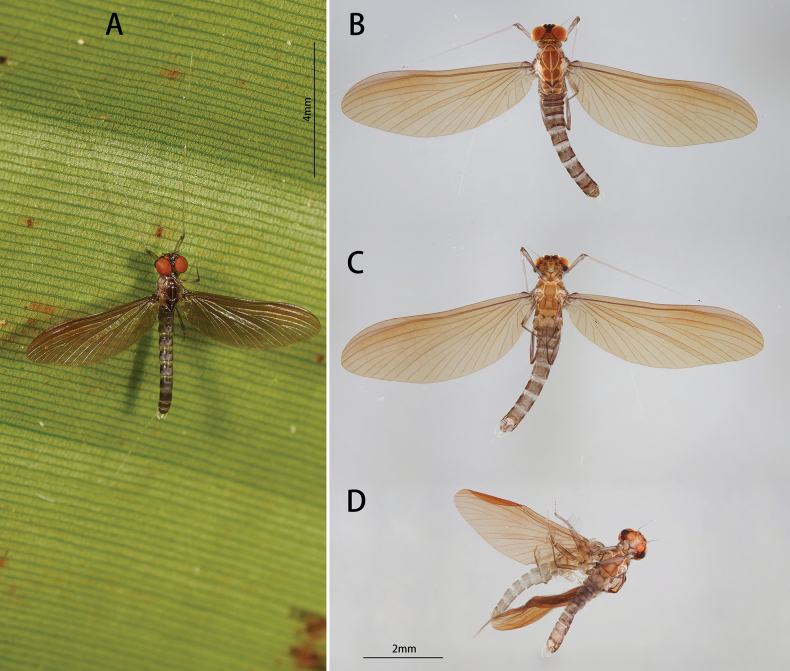
Male of *I.acutata* sp. nov.: **A** imago (dorsal view, living specimen in the field) **B** imago (dorsal view) **C** imago (ventral view) **D** imago with exuviate of subimago.

**Figure 4.**
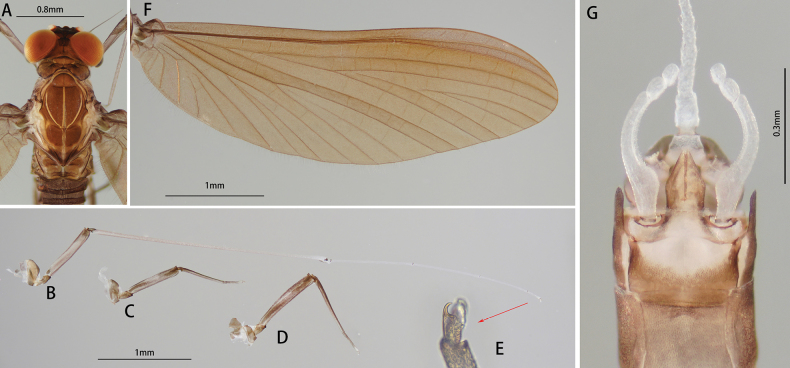
Male structures of *I.acutata* sp. nov.: **A** head (dorsal view) **B** foreleg **C** midleg **D** hindleg **E** claw **F** wing **G** genitalia (ventral view).

**Figure 5.**
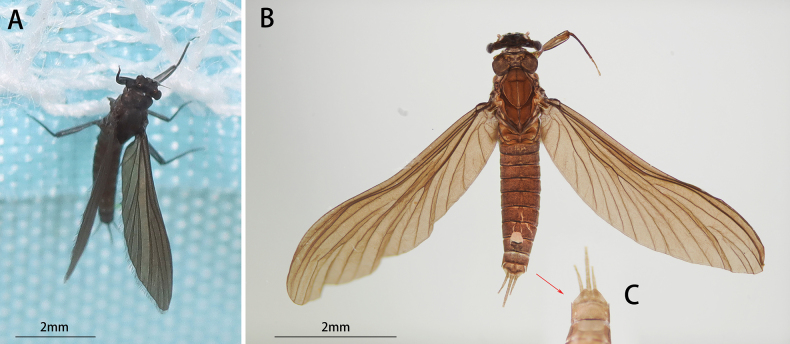
Female structures of *I.acutata* sp. nov.: **A** female subimago (living specimen) **B** female subimago (dorsal view) **C** sternum IX (ventral view).

**Figure 6.**
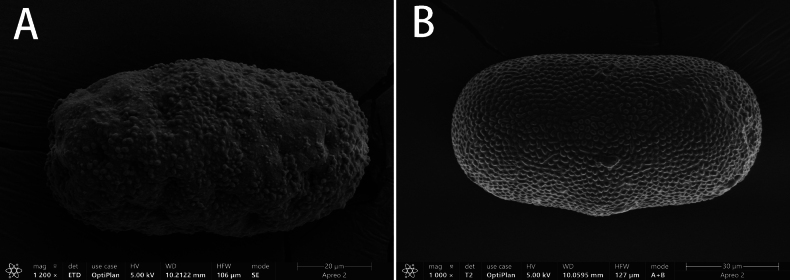
SEM photos: **A** egg from female subimago of *I.acutata* sp. nov. **B** egg from female nymph of *Iscapurpurea*.

Figs 1 , 2 , 3 , 4 , 5 , 6A

##### Material examined.

***Holotype*** • 1 male imago, Shuiman Village, Wuzhishan City, Hainan Province, China, 730m a.s.l., 18.907855°N, 109.679361°E, 2024-I-8-10, Dewen Gong, Xuhongyi Zheng leg. ***Paratypes*** • 1 male imago (reared from nymph) and 10 nymphs, same data as holotype.

##### Other material.

• 3 female subimagoes (reared from nymphs), 21 nymphs, Limu Mountain, Wuzhishan City, Hainan Province, China, 627m a.s.l., 19.169685°N, 109.746047°E, 2023-IV-29, Dewen GONG, Xiaofang Chen, Xinhe Qiang leg. • 4 nymphs, Jianfengling Moutain, Ledong County, Hainan Province, China, 850 m a.s.l., 18.743911°N, 108.854413°E, 2022-VI-29, Dewen Gong, Manqing Ding, Xinhe QIANG leg. • 1 nymph, Nandao Farm, Sanya City, Hainan Province, China, 231 m a.s.l., 18.394447°N, 109.388103°E, 2022-VII-6, Dewen Gong, Manqing Ding, Xinhe Qiang leg. • 2 male imagoes, 5nymphs, Qixianling Mountain, Baoting County, Hainan Province, China, 280 m a.s.l., 18.702331°N, 109.692115°E, 2024-I-11, Dewen Gong, Xuhongyi Zheng leg.

##### Description.

***Nymph (in alcohol)***: Body length 4.2–4.4 mm, cerci subequal to body length, terminal filament 6.4–7.2 mm (Fig. 1A–C). General coloration brown, head and thorax washed light brown, abdominal terga I–VIII brown, terga IX–X light brownish, without any specific pattern, sterna much lighter than terga. Wingpad dark brown, legs uniformly yellowish to amber (Fig. 1A–C).

Head: prognathous, area between three ocelli darker than others (Fig. 1A, C).

Labrum: ca. 1.8 times wider than long, anterior margin concave in the middle, forming a wide V-shape, lateral-anterior angle rounded, lateral-posterior margin shrunken inward; dorsal surface with two rows of setae sub-marginally, setae in the anterior row slender, denser and longer than those in the posterior row, ventral surface with scattered setae near anterolateral angles and a tuft of hair-like setae sub-medially (Fig. 2A).

Mandible: outer margin smoothly convex, with ca. 16 setae at median surface; outer and inner incisors of left mandible divided into three teeth respectively, prostheca with a spur and a tuft of spines; outer incisor of right mandible with 3 teeth (2 tiny denticles on inner tooth), inner incisor with 2 teeth, each of them with additional two acute denticles on both sides; prostheca with a tuft of spines, one bigger than others; four long mesal setae under the molar (Fig. 2B). Hypopharynx: superlinguae with concave lateral margins, two apices extended into oval lobes; apical margin with hair-like setae; two arms of lingua subequal to median oval lobe (Fig. 2C). Maxilla: crown covered with dense of setae, galea-lacinia with row of setae, inner apex with a comb-like setae; maxillary palp three-segmented, length ratio of them from I to III = 1.6: 1.0: 1.0, outer margin of segment I with six setae, segment II with a seta, segment III with two setae, tip of segment III with tuft of relatively short setae; outer margin of stipes with very sparse hair-like setae, outer margin of cardo with six long setae (Fig. 2D). Labium: length ratio of 3 segments of labial palp from I to III = 1.4: 1.1: 1.0; two apical segments slimmer than basal one (Fig. 2E).

Thorax: Setae on outer margin of femur strong, relatively longer than that on inner margin; inner margin of tibiae and tarsi with stout setae; outer margin of tibiae and tarsi with hair-like setae, those on tibia of hindleg with extra stout setae (Fig. 1D–F). Length ratio of femora: tibiae: tarsi of forelegs = 2.3: 1.8: 1.0, that of midlegs = 2.4: 2.0: 1.0, and hindlegs = 2.5: 2.3: 1.0 (Fig. 1D–F). Claw with 14 denticles, distal one larger than others, proximal four denticles smaller (Fig. 1G).

Abdomen: posterior margin of each tergite with contiguous acute spines, posterolateral projections on segment IX only (Fig. 1A–C). Gills present on segments II to VII of ventral side of abdomen (Fig. 1B). Gills similar morphologically, consist of two slender and unbranched lamellae, gills progressively smaller from anterior to posterior (Fig. 1H–I). Caudal filaments brown, terminal filament slightly darker than cerci; posterior margin of each segment with spines, every second segment from segment II with an encircling row of hair-like setae around posteriorly (Fig. 1A–C).

***Male imago (in alcohol)***: Body length 4.4 mm, generally amber to brownish, wings deep brown (Fig. 3).

Head: Width between two compound eyes about half of one eye width; upper half of it orange, lower half black (Fig. 4A).

Thorax: Legs: Foretarsus and tip of tibia pale, other portion brown, ratio of femur: tibia: tarsus = 1.0: 3.8: 3.5, ratio of foretarsal segments from I to V = 1.0: 14.0: 11.9: 8.6: 2.5. Midleg and hindleg dark brown, ratios of femur: tibia: tarsus = 2.9: 2.4: 1.0 and 2.9: 2.5: 1.0 (Fig. 4B–D). Claw dissimilar, one hooked, one blunt (Fig. 4E).

Wing opaque, brown; veins clear, posterior margin with cilia, vein MA forked slightly less than 1/2 of distance from base to margin (Fig. 4F).

Abdomen: color of terga I to IX alike, anterior 1/3 pale and semi-transparent, posterior 2/3 brown, sterna lighter than terga; tergite X brown (Fig. 3). Genitalia: forceps pale; segment I with a mesal expansion at 2/5 point; segments II and III subequal, nearly sphere; penes brown, two penes situated together, forming a pen-point like structure, margin of basal half straight, apical half straightly oblique; length of penes about 2/5× of forceps (Fig. 4G). Caudal filaments pale (Figs 3A, 4G).

**Female**: body length 4.1 mm, caudal filaments brown with spine-like setae. Coloration similar to male, sternum IX slightly concave apically (Fig. 5).

##### Egg.

Egg oval, length about 85 μm, width about 45 μm, surface scattered with protuberances and cavities; their size and location irregular (Fig. 6A).

##### Biology.

**Figure 7.**
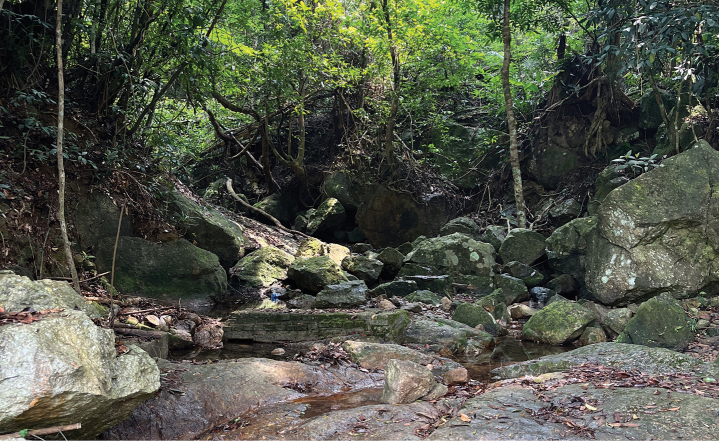
Habitat of *I.acutata* sp. nov.

Nymphs found under stones with coarse surfaces in slow water currents of a montane stream about 1–2 m, without direct sunlight (Fig. 7). Adults emerged in the late afternoon. Some male imagoes were found under leaves above the stream.

##### Etymology.

*acutata* comes from the Latin adjective *acutatus*, indicating the shape of sharp penal apex of the new species.

##### Diagnosis.

The nymph of *Iscaacutata* sp. nov. can be distinguished from other *Isca* species by the following characters: abdominal terga I–VIII brown, terga IX–X light brown (Fig. 1A, C); abdominal sterna light brown (Fig. 1B); gill VII with dorsal and ventral lamella (Fig. 1I); posterolateral projection on segment IX (Fig. 1B). The male imago of *I.acutata* sp. nov. can be distinguished by: wings translucent with cilia along posterior margin; anterior 1/3 of each abdominal tergum, and sterna I–VIII pale and semi-transparent; posterior 2/3 brown, sterna slightly lighter than terga (Fig. 3); forceps pale; penes brown, fused basally, nib-like (Fig. 4G).

##### Comparison.

Among six known species, only two species (*I.lea* and *I.acutata* sp. nov.) have bilamellate gills VII in their nymphal stage. Those two nymphs can be differentiated by: (1) ventral setae on labrum of *Iscaacutata* sp. nov. is less than *Iscalea* because it has two setal tufts (Fig. 2A), and (2) more setae on the tip of the maxillary papa segment III of *Iscaacutata* than *Iscalea* (Fig. 2D).

In males, the new species *Iscaacutata* sp. nov. is unique in penal shape: penes fused basally (Fig. 4G) while those of *I.purpurea*, *I.serendiba*, *I.janiceae* are separated; its apical margin is straight (Fig. 4G) while that of *I.fascia* is convex (Fig. 15C); vein MA is forked less than 1/2 of distance from base to margin (Fig. 4F) while vein MA of *I.janiceae* and *I.purpurea* forked more than 1/2 of distance from base to margin; cilia present on posterior margin of wings (Fig. 4F) while absent in *I.janiceae*; sternum IX of female *Iscaacutata* sp. nov. cleft (Fig. 5C) but not as deeply as *I.purpurea* (Fig. 12B), while that of *I.janiceae* is entire.

##### Distribution.

China (Hainan Island).

#### ﻿ Isca purpurea

Taxon classificationAnimaliaEphemeropteraLeptophlebiidae

Gillies, 1951

BADF030C-CCB7-5F0D-ABCE-D2A0225289F9

**Figure 8.**
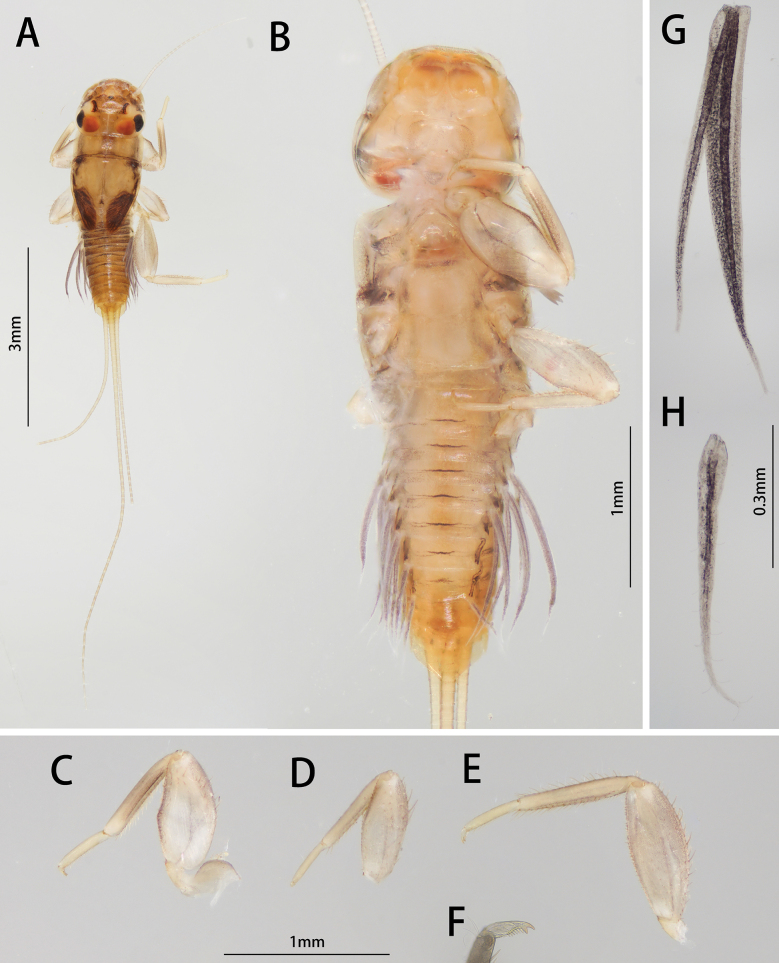
Nymphal structures of *I.purpurea*: **A** male nymph (dorsal view) **B** male nymph (ventral view) **C** foreleg **D** midleg **E** hindleg **F** claw **G** gill IV **H** gill VII.

**Figure 9.**
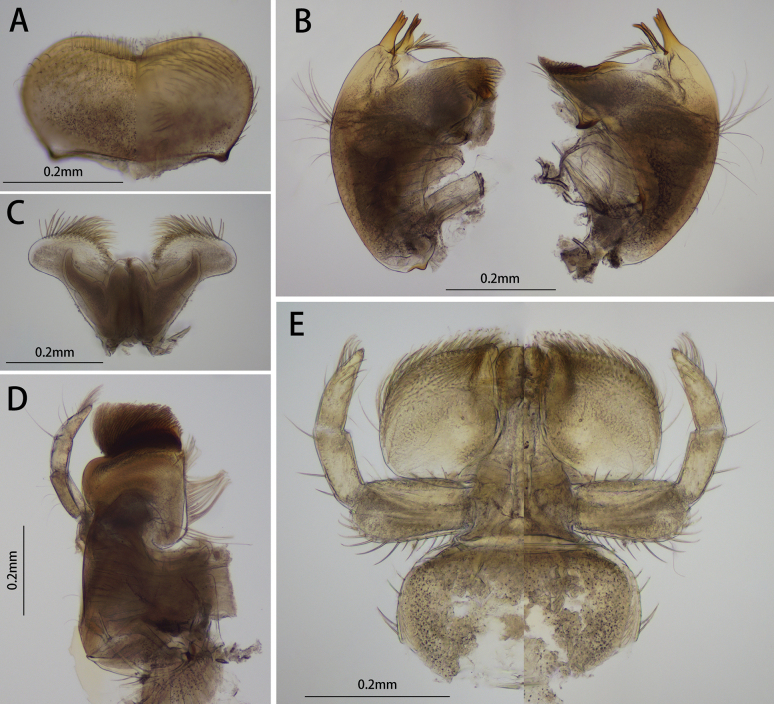
Mouth part of *I.purpurea*: **A** labrum (dorsal view) **B** mandible (left: left mandible / right: right mandible) **C** hypopharynx **D** maxilla (ventral view) **E** labium (left: dorsal view / right: ventral view).

**Figure 10.**
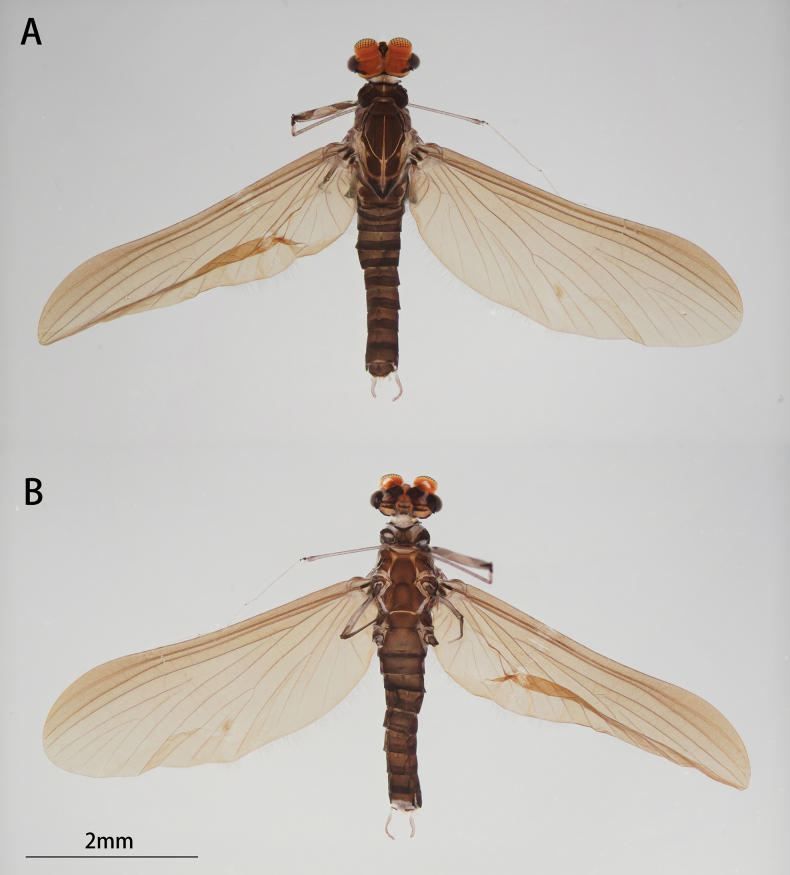
Male imago of *I.purpurea*: **A** dorsal view **B** ventral view.

**Figure 11.**
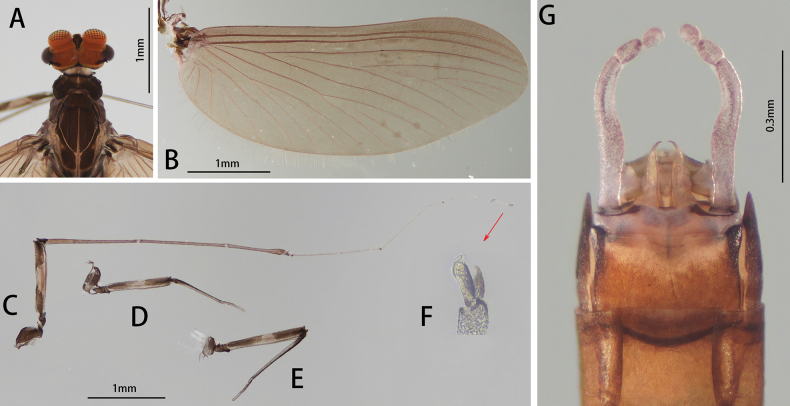
Male structures of *I.purpurea*: **A** head (dorsal view) **B** wing **C** foreleg **D** midleg **E** hindleg **F** claw **G** genitalia (ventral view).

**Figure 12.**
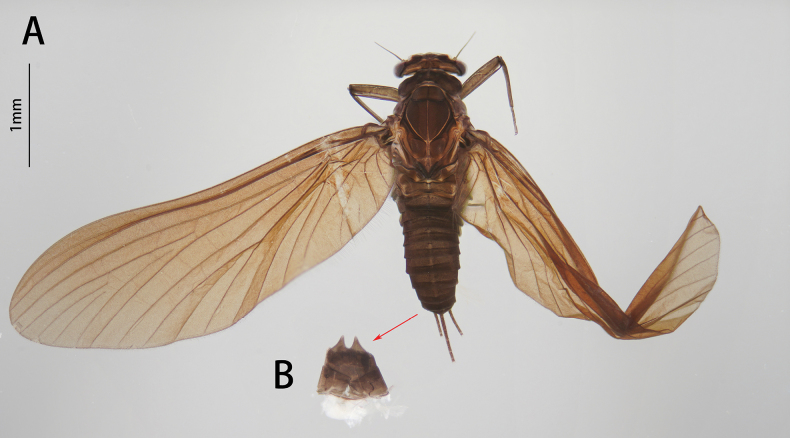
Female structures of *I.purpurea*: **A** female subimago (dorsal view) **B** sternum IX (ventral view).

Figs 6B , 8 , 9 , 10 , 11 , 12

Isca purpurea Gillies, 1951: 21–130, figs 15–22 (male, female). Type: male, from China (Hong Kong) and India (Mirik).
Isca purpurea : Peters and Edmunds 1970: 159–240, figs 71, 106, 330, 350–357 (nymph, male).

##### Material examined.

• 55 nymphs, Chebaling Mountain, Shaoguan City, Guangdong Province, China, 460 m a.s.l., 24.701644°N, 114.190687°E, 2024-IV-2-14, Dewen Gong, Xuhongyi Zheng leg. • 10 male imagoes, 3 female subimagoes, 3 nymphs, Laipoli, Jinan District, Fuzhou City, Fujian Province, China, 390 m a.s.l., 26.251947°N, 119.283899°E, 2024-IV-25, Xuhongyi Zheng leg. • 5 nymphs, Jiulian Mountain, Ganzhou City, Jiangxi Province, China, 24.539800°N, 114.467900°E, 2020-IX-14, Zhengxing MA leg.

##### Description.

***Nymph (in alcohol, first formal description)***: Body length 4.0–4.5 mm (Fig. 8A, B).

General coloration brownish, thorax light brown, abdomen brownish, without any specific pattern, ventral surface much lighter. Wingpad dark brown, legs uniformly yellowish (Fig. 8A, B).

Head: prognathous (Fig. 8A, B). Labrum ca. 1.8 times wider than long, anterior margin concaved in the middle, lateral-anterior angle rounded, lateral-posterior margin shrunken inward; dorsal side with two rows of setae anteriorly, anterior row denser than posterior row but length of them subequal (Fig. 9A).

Mandible with rounded outer margin, with 14 setae in the middle; outer incisor of left mandible with 3 apical teeth, inner incisor with 3 teeth, prostheca with a spur and a tuft of spines; outer incisor of right mandible with 3 teeth, inner incisor with 2 teeth and a denticle aside, prostheca with a relatively small spur and a tuft of spines, 4 long setae under the molar (Fig. 9B).

Hypopharynx: superlinguae with concave lateral margins, apex with setal tufts, lateral angles rounded (Fig. 9C).

Maxilla covered with dense crown of setae, galea-lacinia with row of setae, margin of cardo with 5 long setae; maxillary palp 3-segmented, length ratio of 3 segments from I to III = 1.6: 1.0: 1.2, outer margin of segment I with 5 setae, segment II with a seta on the tip, segment III with 2 setae, tip of segment III with tuft of relatively short setae (Fig. 9D).

Labium like other *Isca* species, the ratio of 3 segments of labial palp from I to III = 1.3: 0.9: 1.0 (Fig. 9E).

Thorax: Setae on outer margin of femur strong, relatively longer than that on inner margin; inner margin of tibiae and tarsi with stout setae; outer margin of tibiae and tarsi with hair-like setae, those on tibia of hindleg with extra stout setae (Fig. 8C–E). Claw with 11 denticles 4 on inner side small, 1 on outer side biggest (Fig. 8F). Ratio of femora: tibiae: tarsi of forelegs = 2.0: 1.8: 1.0, that of midlegs is 2.3: 1.9: 1.0, and hindlegs = 2.5: 2.2: 1.0 (Fig. 8C–E).

Abdomen: posterior surface of each tergite with contiguous acute spines, posterolateral projection on segment IX only (Fig. 8A, B). Gills present on segments II to VII of ventral abdomen (Fig. 8B). Gills II to VI similar morphologically, consisted of two slender and unbranched lamella, gill VII with single slender lamella (Fig. 8G, H).

***Male imago*** (in alcohol; also see Gillies 1951 and Peters and Edmunds 1970):

Body length 4.5 mm, generally brownish (Fig. 10). Head: Compound eyes contiguous basally, upper part separated with dark stripes around facets (Fig. 11A).

Legs generally dark brown, mid part of femora slightly lighter than both ends, tarsus of foreleg brownish from base to tip (Fig. 11C). Ratio of femur: tibia: tarsus of foreleg = 1.0: 3.1: 3.1, those of midleg and hind leg = 2.9: 2.2: 1.0 and 3.2: 2.5: 1.0, ratio of foretarsal segments from I to V = 1.0: 13.7: 12.5: 8.0: 3.0 (Fig. 11C–E). Claw dissimilar, one hooked and acute, one blunt (Fig. 11F).

Wing brown, transparent; veins clear, posterior margin with cilia, vein MA forked slightly more than 1/2 of distance from base to margin (Fig. 11B).

Abdomen: tergites of both dorsal and ventral view dark brown; tergite IX with posterolateral projection (Fig. 10). Genitalia: forceps brownish, segment I longest, midpart slightly protuberant inward; segment II slightly longer than III. Penes brownish; broad at base, gradually narrowing to tip; base separated; tips sharp, bend inward; length of penes about 2/5 of forceps (Fig. 11G).

***Female (in alcohol)***: Body length 4.2 mm, terminal filaments brown with spine-like setae. Coloration like male, apex of sternum IX deeply concave (Fig. 12).

##### Egg.

Egg oval, length about 100 μm, width about 50 μm, surface covered with subequal small protuberances (Fig. 6B).

##### Biology.

**Figure 13.**
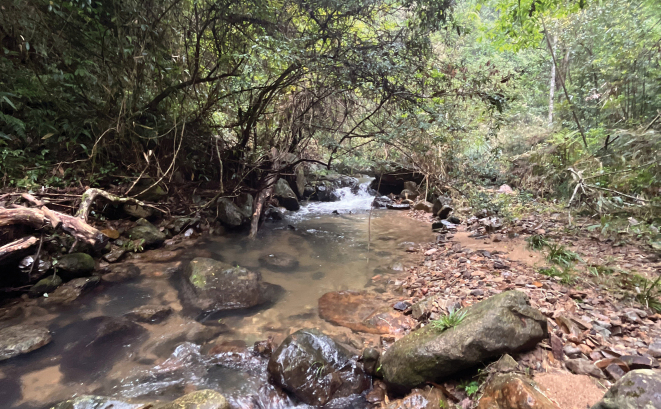
Habitat of *I.purpurea*.

Like *I.acutata*, nymphs of *I.purpurea* were found under stones with coarse surfaces in slow flowing mountain stream; the stream was about 2 m wide with good shade (Fig. 13).

##### Diagnosis.

The nymph of *I.purpurea* can be distinguished from other *Isca* species by the following characters: abdominal terga brown, and abdominal sterna light brown. The male imago of *I.purpurea* can be distinguished by vein MA forked slightly more than 1/2 of the distance from base to margin (Fig. 11B); cilia present on the posterior margin of the wings (Fig. 11B); abdominal terga and sterna dark brown (Fig. 10); forceps and penes brown (Fig. 11G); and penes separated widely at base, tip hook-like, curved inward (Fig. 11G).

##### Distribution.

China (Hong Kong, Guangdong, Fujian, Jiangxi); India.

#### ﻿ Isca fascia

Taxon classificationAnimaliaEphemeropteraLeptophlebiidae

Nguyen & Bae, 2003

AF355A1E-D144-59A2-9EBE-C1973EAF9794

**Figure 14.**
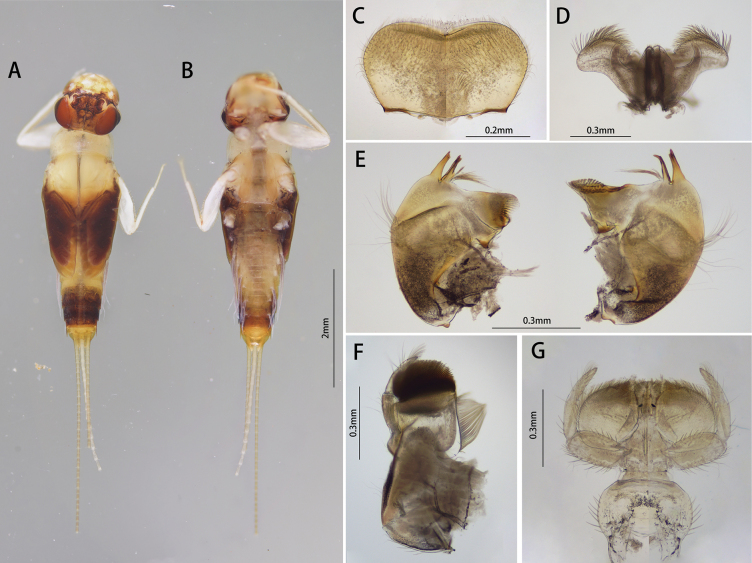
Nymphal structures of *I.fascia*: **A** male nymph (dorsal view) **B** male nymph (ventral view) **C** labrum (left: dorsal view, right: ventral view) **D** hypopharynx **E** mandible (left: left mandible, right: right mandible) **F** maxilla (ventral view) **G** labium (left: dorsal view, right: ventral view).

**Figure 15.**
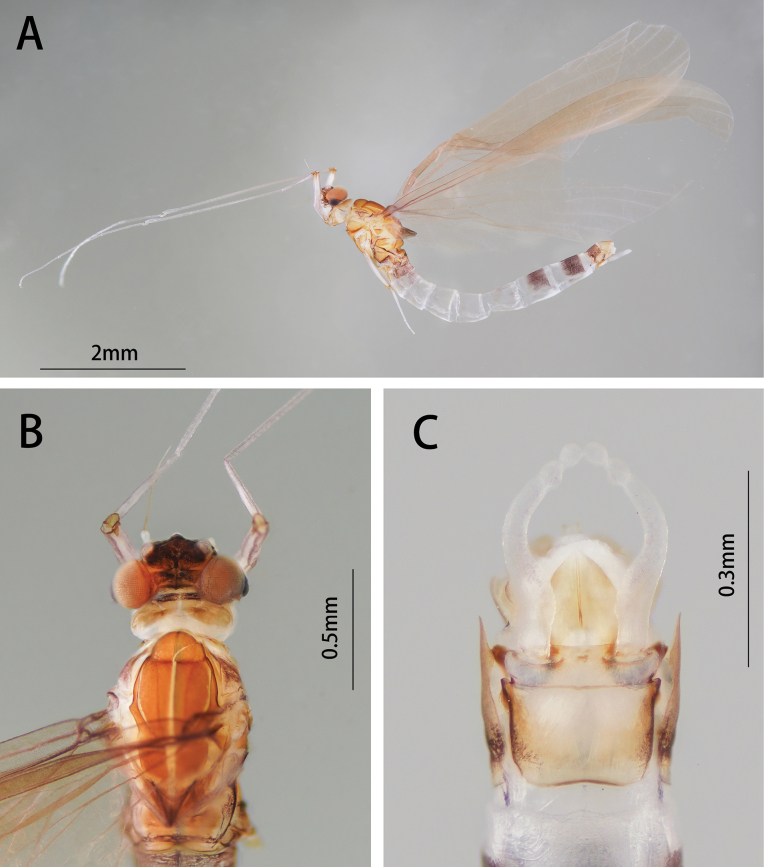
Male structures of *I.fascia*: **A** male imago (lateral view) **B** head (dorsal view) **C** genitalia (ventral view).

Figs 14 , 15 (first record from China)

Isca fascia Nguyen & Bae, 2003: 453–466, figs 20–24 (nymph, male). Type: female nymph, from Vietnam.

##### Material examined.

• 1 male imago 11 nymphs, Ailao Mountain, Yuxi City, Yunnan Province, China, 2200 m a.s.l., 23.970333°N, 101.527147°E, 2022-II-7, Xuhongyi Zheng leg.

##### Description.

(see Nguyen and Bae 2003).

##### Diagnosis.

The nymph of *I.fascia* can be distinguished from other *Isca* species by the following characters: abdominal terga I–II and VII–IX dark brown, terga III–VI light brown (Fig. 14A); abdominal sterna I–VI and X light brown, sterna VII–IX dark brown (Fig. 14B). The male imago of *I.fascia* can be distinguished by: wings translucent, vein MA forked slightly more than 1/2 of distance from base to margin; cilia present on posterior margin of wings (Fig. 15A); abdominal terga I–II and VII–IX dark brown, terga III–VI pale (Fig. 15A); forceps pale (Fig. 15C); penes yellow, large, lateral margin round, fused basally (Fig. 15C).

##### Distribution.

China (Yunnan Prov.); northern Vietnam.

### ﻿Genetic distance

Both materials of Chinese *I.purpurea* and *I.acutata* sp. nov. have very close genetic distance. The COI K2P distance of *I.purpurea* is from 0.29% to 1.94%, while that of *I.acutata* sp. nov. is 0.48% (Table 2). So, our morphological identification is supported by genetic distance.

Similarly, three Indian *I.purpurea* populations have short COI sequence distances, which are 1.05% and 11.81% respectively. However, the Chinese *I.purpurea* and Indian *I.purpurea* have relatively large genetic distances: the K2P distance is from 16.47% to 20.12% (Table 2).

## ﻿Discussion

**Figure 16.**
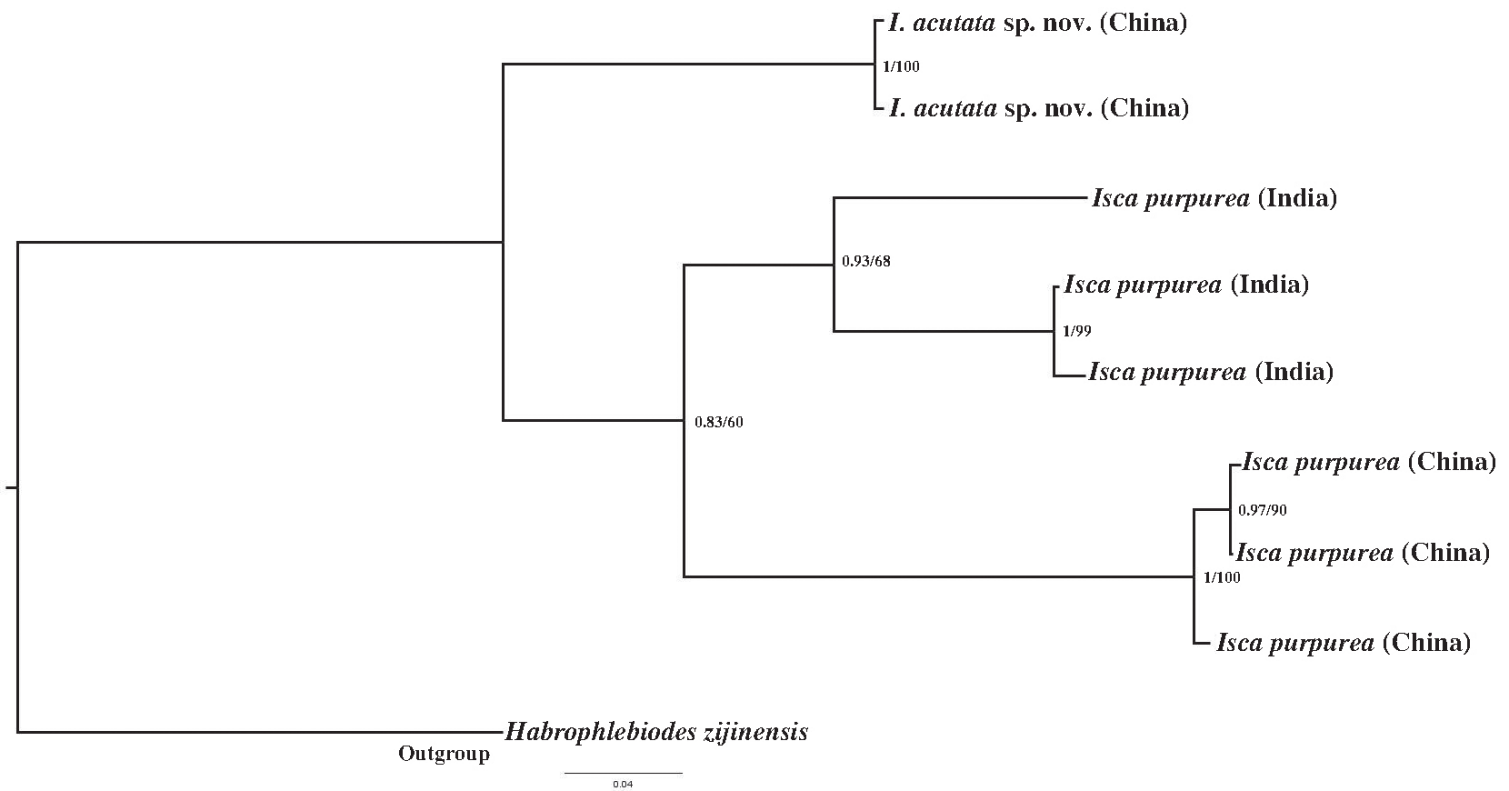
the BI and MP tree upon COI sequences of Indian and Chinese *Iscapurpurea*.

Gillies (1951) designated the specimens from Hong Kong as types and mentioned there are differences between Indian and Chinese material of *I.purpurea*, stating the Bengal specimen has rather darker eyes. Here we compared the COI sequences of Indian specimens (GenBank: MW160191, ON557588, LC061468, Table 1) and our specimens. The K2P distance of those two populations is from 16.47% to 20.12% (Table 2). This point is reflected in the BI and MP tree (Fig. 16), in which the Chinese *Iscapurpurea* and Indian *Iscapurpurea* are clustered into two different clades. So, the Indian “*Iscapurpurea*” needs more work to confirm its real status.

Considering these above, together with the huge distance between India and China, we propose that the Indian and Chinese species of *Iscapurpurea* could be classified as separate species. Despite certain similarities, further studies are needed to clarify their distinctions (Gillies 1951).

**Figure 17.**
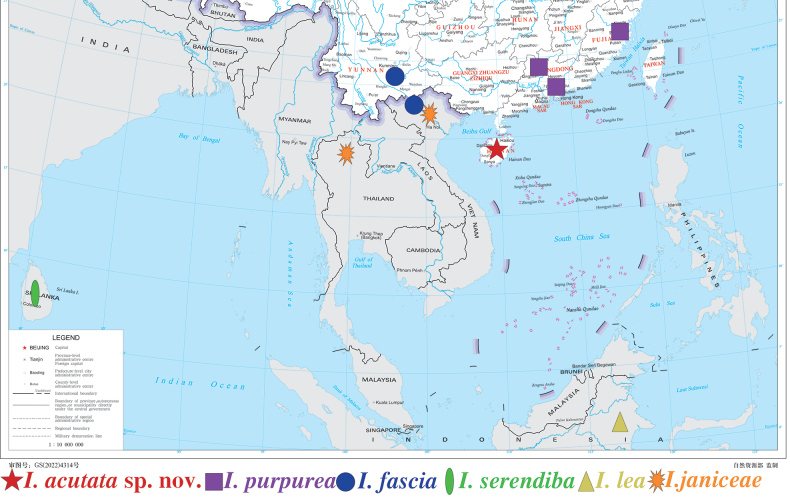
Distribution of *Isca* spp.

Based on previous research and our study, the distribution of *Isca* spp. is now given (Fig. 17). It is shown that South China has a relatively rich species diversity of genus *Isca*, and we believe South Asia and Southeast Asia contain many more *Isca* species.

Gillies (1951) referred to “subimago” and “subimagines” but did not specify their sexes. Peters and Edmunds (1970) and Nguyen and Bae (2003) did not mention or describe the subimago stage at all. Kluge (2014) noted that “the *Isca* female subimago does not molt to imago.” In our observations, male adults exhibit both subimaginal and imaginal stages, whereas the female’s subimago represents its final stage. During our rearing process, three female subimagoes of the new species, *I.acutata* sp. nov., successfully emerged from nymphs without undergoing any further molts. In contrast, one male individual was able to molt twice from nymph to imago, with the final molt occurring 2–3 hours after it reached the subimago stage. Additionally, the morphology of the female adult, particularly their cerci, is similar to that of the male subimago. Moreover, the female adults of *I.purpurea*, collected from cobwebs and exhibiting empty or nearly empty abdomens, resemble the females of *I.acutata* sp. nov.

The new species, *I.acutata* sp. nov., found in Hainan, is notable as the second species to possess two lamellae on gill VII. Apart from this characteristic, there are no other obvious differences in the nymphs or adults when compared to other known species. Furthermore, the reduction or loss of gills has occurred multiple times across various lineages within Ephemeroptera. Therefore, we consider the presence of bilamellate gills to be a useful diagnostic character, albeit not an essential synapomorphy.

Peters and Edmunds (1970) divided the genus *Isca* into three subgenera (*Isca*, *Minyphlebia*, *Tanycola*) upon imaginal characteristics. However, *I.fascia* has mixed characteristics of those subgenera: 1) like *Minyphlebia*, vein MA forked less than 1/2 of distance from base to margin (Fig. 15A); 2) like *Isca* and *Tanycola*, cilia are present on the posterior margin of the wings (Fig. 15A); and 3) penes are fused basally (Fig. 15C), a unique character, not reported in any subgenera. Under this circumstance, Nguyen and Bae (2003) and Sartori and Derleth (2010) did not place their species *I.fascia* or *I.lea* into any subgenus. Here, the new species *I.acutata* sp. nov. also has the same imaginal characteristics as *I.fascia* and remarkable bilamellate gills VII. So, at this moment, we have not placed our three species into any subgenus here.

## Supplementary Material

XML Treatment for Isca acutata

XML Treatment for Isca purpurea

XML Treatment for Isca fascia

## ﻿Additional information

### Author ORCIDs

De-Wen Gong https://orcid.org/0000-0002-3740-7125

Chang-Fa Zhou https://orcid.org/0000-0001-8785-5228

### Data availability

All of the data that support the findings of this study are available in the main text.
